# P-491. Optimizing Delivery of HIV Pre-Exposure Prophylaxis (PrEP) to Adolescents at Risk of HIV Infection

**DOI:** 10.1093/ofid/ofaf695.706

**Published:** 2026-01-11

**Authors:** Charlotte Formeller, Wendi Ehrman, Margaret Thew, Lia Mojica, Claudia P Vicetti Miguel

**Affiliations:** Medical College of Wisconsin, Milwaukee, WI; Medical College of Wisconsin 8701 W. Watertown Plank Rd, Milwaukee, WI 53226, Milwaukee, Wisconsin; MCW, Milwaukee, Wisconsin; Medical College of Wisconsin, Milwaukee, WI; Medical College of Wisconsin, Milwaukee, WI

## Abstract

**Background:**

The CDC and AAP recommend HIV Pre-exposure Prophylaxis (PrEP) be discussed with all sexually active adolescents. As of 2021, 19% of new HIV diagnoses in the US were among youth ages 13-24. In our city of Milwaukee, this age group accounted for 30% of new cases. Our objectives were to identify barriers to PrEP prescribing among pediatric primary care providers (PCPs), implement targeted interventions, and evaluate their impact on rates of PrEP prescribing over 24 months.

**Figure 1:**
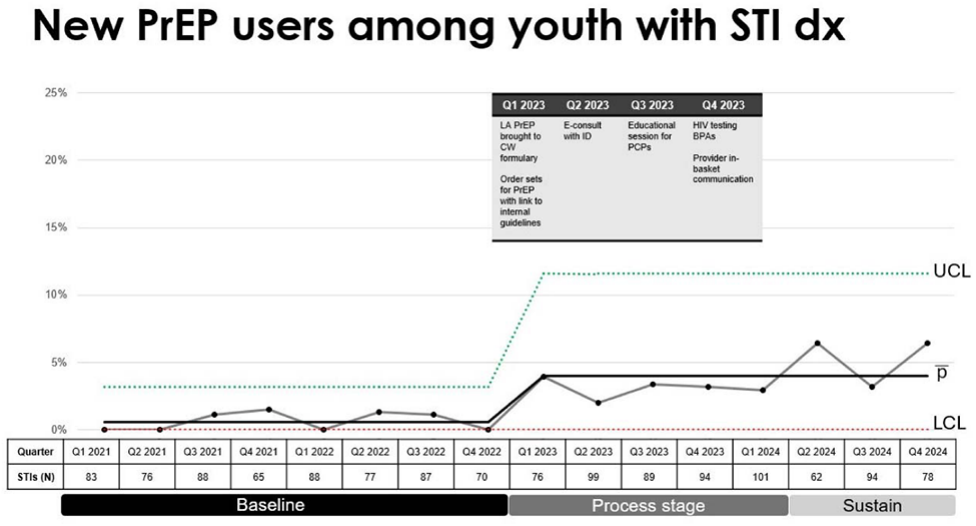
Statistical process control chart of new HIV PrEP prescriptions in youth with an STI diagnosis. Baseline data collected Jan 2021-Dec 2022, process stage from Jan 2023-Mar 2024 with implementation of each intervention listed by quarter, along with post-intervention stage from Apr 2024-Dec 2024 showing a sustained increase in prescriptions from baseline.

**Methods:**

This quality improvement project surveyed PCPs affiliated with a tertiary non-for-profit children's hospital serving southeastern Wisconsin regarding PrEP prescribing to identify common barriers. Interventions included adding injectable PrEP to formulary, an educational session, and EMR tools to identify eligible patients and support prescribing. PrEP initiation among adolescents at risk (defined as those with a bacterial STI) were monitored during baseline (Jan 2021-Dec 2022) and post-intervention periods (Jan 2023-Dec 2024) using Plan-Do-Study-Act (PDSA) cycles tracked via a statistical process control chart.

**Results:**

Of the PCPs surveyed, 93% (55/59) had never prescribed PrEP; 51% were not aware of its’ indications, and 24% were uncomfortable with its’ management. The educational session reached 83/131 (63%) PCPs, increasing prescribing comfort from 36% to 100% in pre and post session assessments, respectively. HIV PrEP prescribing rates increased from 0.6% (4/634) of adolescents with an STI to 3.8% (26/693) post-intervention. PrEP counseling rates increased from 1.4% to 12% after the intervention period (Figure 1). All new HIV PrEP patients had appropriate baseline testing and timely follow up.

**Conclusion:**

Most PCPs are not prescribing PrEP due to lack of awareness and comfort. Implementation of provider education session, EMR tools, and availability of injectable PrEP led to sustained impact on provider confidence, HIV counseling frequency, and HIV PrEP prescribing rates.

**Disclosures:**

Lia Mojica, PIDS SUMMERS Program: Award recipient

